# Discovery of Cinnamylidene Derivative of Rhodanine with High Anthelmintic Activity against *Rhabditis* sp.

**DOI:** 10.3390/molecules27072155

**Published:** 2022-03-27

**Authors:** Waldemar Tejchman, Przemysław Kołodziej, Justyna Kalinowska-Tłuścik, Wojciech Nitek, Grzegorz Żuchowski, Anna Bogucka-Kocka, Ewa Żesławska

**Affiliations:** 1 Institute of Biology, Pedagogical University of Krakow, Podchorążych 2, 30-084 Kraków, Poland; waldemar.tejchman@up.krakow.pl; 2 Chair and Department of Biology and Genetics, Faculty of Pharmacy, Medical University of Lublin, Chodźki 4a, 20-093 Lublin, Poland; przemyslaw.kolodziej@umlub.pl (P.K.); anna.kocka@umlub.pl (A.B.-K.); 3 Faculty of Chemistry, Jagiellonian University, Gronostajowa 2, 30-387 Kraków, Poland; justyna.kalinowska-tluscik@uj.edu.pl (J.K.-T.); wojciech.nitek@uj.edu.pl (W.N.); 4 Department of Organic Chemistry, Faculty of Pharmacy, Jagiellonian University Medical College, Medyczna 9, 30-688 Kraków, Poland; grzegorz.zuchowski@uj.edu.pl

**Keywords:** cinnamylidene derivatives of rhodanine, anthelmintic activity, crystal structure, molecular modelling, *Rhabditis* sp.

## Abstract

The treatment of parasitic infections requires the application of chemotherapy. In view of increasing resistance to currently in-use drugs, there is a constant need to search for new compounds with anthelmintic activity. A series of 16 cinnamylidene derivatives of rhodanine, including newly synthesized methoxy derivatives (**1**–**11**) and previously obtained chloro, nitro, and diethylamine derivatives (**12**–**16**), was investigated towards anthelmintic activity. Compounds (**1**–**16**) were evaluated against free-living nematodes of the genus *Rhabditis* sp. In the tested group of rhodanine derivatives, only compound **2** shows very high biological activity (LC_50_ = 0.93 µg/µL), which is higher than the reference drug albendazole (LC_50_ = 19.24 µg/µL). Crystal structures of two compounds, active **2** and inactive **4**, were determined by the X-ray diffraction method to compare molecular geometry and search for differences responsible for observed biological activity/inactivity. Molecular modelling and selected physicochemical properties prediction were performed to assess the potential mechanism of action and applied in the search for an explanation as to why amongst all similar compounds only one is active. We can conclude that the tested compound **2** can be further investigated as a potential anthelmintic drug.

## 1. Introduction

Multidrug resistance (MDR) has become a factor seriously limiting the treatment of various diseases, including parasitic infections [1]. Parasitic infections are connected with the living environment and are widely distributed in tropical and subtropical areas. They are not transmitted directly from person to person. In the case of malaria, parasites are transmitted to people through the bites of infected mosquitoes. According to WHO’s estimation, in 2020 there were about 241 million cases of malaria infections worldwide, which led to 627 thousand deaths [2]. Soil-transmitted helminth infections caused by different species of parasitic worms are also a big problem in the modern world. They are transmitted by eggs present in human faeces, which lead to contamination of the soil in areas where sanitation is poor. As reported by WHO, about 1.5 billion people are infected with soil-transmitted helminth infections [3]. More than half of those infected are children. Infections can cause anaemia, intestinal manifestations, general malaise and weakness, and impaired growth and physical development. Chemotherapy is the main direction to control and fight against parasitic infections [4]. Thus, it is crucial to search for new anthelmintic drugs or other strategies allowing to overcome MDR. 

**Figure 1:**
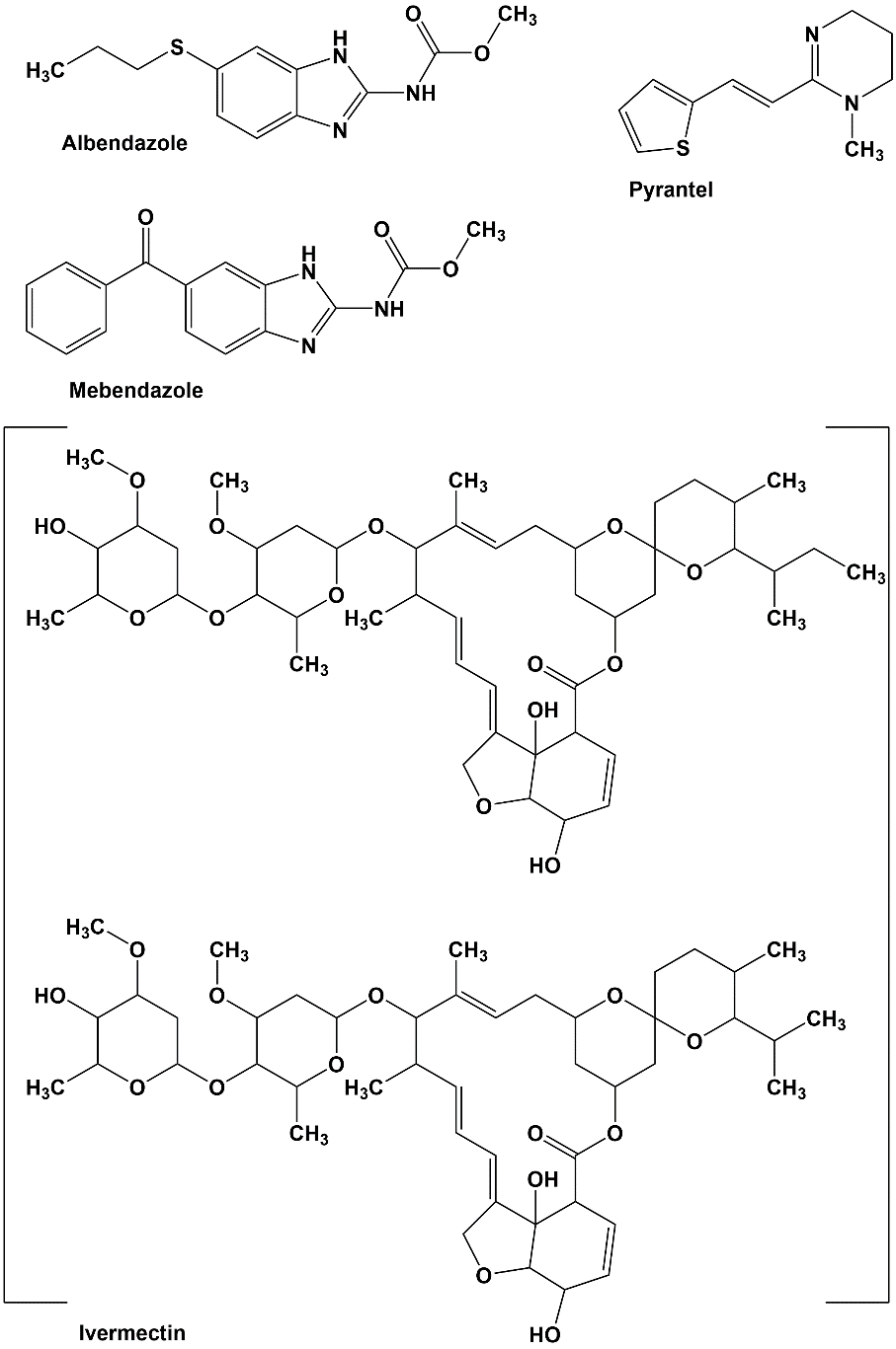
The anthelmintic drugs exhibiting nematicidal activity.

In the group of anthelmintic drugs there are benzimidazoles (albendazole, mebendazole and flubendazole), salicylanilides (niclosamide, rafoxanide, closantel), imidazothiazoles (levamizole), thiazolides (nitazoxanide), macrocycliclactones (ivermectin), antitrematodals (praziquantel), quinolines (pyrvinium), cyclic amidines derivatives (pyrantel) [5,6]. Among these above-mentioned compounds, there is a small group with nematicidal activity such as: albendazole, mebendazole, ivermectin and pyrantel (Figure 1). Albendazole and mebendazole change the metabolism of parasites, while ivermectin and pyrantel paralyze the parasite, which leads to their death [6,7,8]. 

Our interest is focused on rhodanine (2-sulfanylidene-1,3-thiazolidin-4-one) derivatives, which were synthesized in our laboratory and tested for their biological activities [9,10,11,12,13]. Derivatives of rhodanine show a broad spectrum of biological activities, e.g., antibacterial, antifungal, antitubercular, anticancer, antidiabetic, anti-inflammatory and antiparasitic activities [14,15,16,17,18,19,20,21,22]. Given the high potency of rhodanine derivatives as bioactive agents, we have evaluated cinnamylidene derivatives of rhodanine towards their anthelmintic activity. At the first stage were tested 3-rhodanine acetic acid and 3-rhodanine propionic acid derivatives containing various substituents at the aromatic ring of the cinnamylidene moiety, namely methoxy (**2**, **3**), nitro (**12**), chloro (**13**, **14**) and N,N-diethyl (**15**, **16**) (Table 1). In the group of investigated compounds, only compound **2** showed anthelmintic activity against *Rhabditis* sp. In this context, we have decided to synthesize other 5-cinnamylidene derivatives of rhodanine containing a methoxy substituent at the aromatic ring in positions *ortho* or *meta* (**8**–**11**), as well as derivatives containing a methoxy substituent in the *para* position without carboxyl group (**1**), followed by compounds containing a carboxyl group with different lengths of the linker (**4**–**7**). The newly synthesized compounds were also evaluated against anthelmintic activity using free-living nematodes of the genus *Rhabditis* sp. According to the available literature, these nematodes can cause *inter alia* outer ear canal and urinary tract infections. Furthermore, *Rhabditis* sp. were also found in children [23,24,25,26,27,28].

Thus, this paper presents the synthesis of a series of new compounds (**1**–**11**) and structural analysis with X-ray crystallography for two selected compounds (**2** and **4**). Furthermore, the newly synthesized compounds (**1**–**11**) and these synthesized earlier (**12**–**16**) [10] were evaluated with regard to their anthelmintic activity. The bioactivity was observed only for one compound (**2**), which prompted us to search further for the explanation of the obtained results. We have analyzed the molecular geometries and intermolecular interactions of active compound **2** and inactive **4** in the crystals in order to search for structural features and geometrical parameters which could be responsible for the biological activity. Additionally, molecular docking studies have been performed to examine the putative mechanism of action and selected physicochemical properties have been estimated to find the plausible explanation as to why only one compound of the whole studied series exhibits the desired bioactivity.

## 2. Results and Discussion

### 2.1. Synthesis

**Scheme 1:**
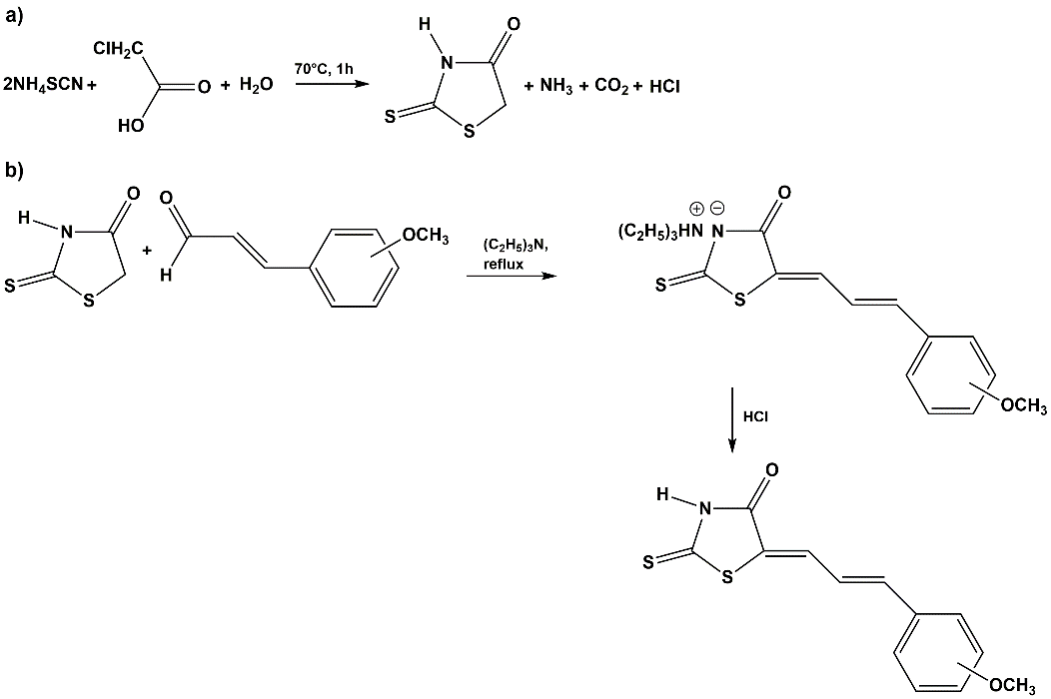
Synthetic route for compounds **1**, **8** and **10**: (**a**) synthesis of rhodanine and (**b**) condensation of rhodanine with an aldehyde.

**Scheme 2:**
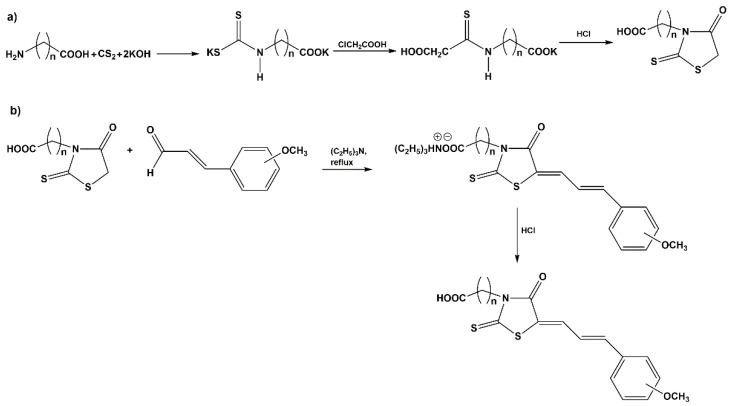
Synthetic route for compounds **2**–**7**, **9** and **11**: (**a**) synthesis of rhodanine-3-carboxyalkyl acids and (**b**) condensation of rhodanine-3-carboxyalkyl acid with an aldehyde.

The final compounds **1**–**11** were obtained within two-step synthesis routes (Scheme 1b and Scheme 2b) based on the procedures described previously for compounds **12**–**16** [10]. The synthetic pathway also required obtaining the rhodanine (Scheme 1a) and the carboxyalkylrhodanine acids (Scheme 2a). In the first step, the rhodanine and cinnamaldehyde derivatives were used to obtain the triethylammonium salts of final compounds **1**, **8** and **10** (Scheme 1b), which were reacted with hydrochloride acid to form the corresponding final products. In the case of other compounds (**2**–**7**, **9** and **11**), the corresponding carboxyalkylrhodanine acid and cinnamaldehyde derivatives were reacted to access the intermediates, namely the triethylammonium salts. Next, hydrochloride acid was added in order to obtain the corresponding final products.

### 2.2. X-ray Crystallographic Studies

#### 2.2.1. Crystal Structures of **2** and **4**

**Figure 2:**
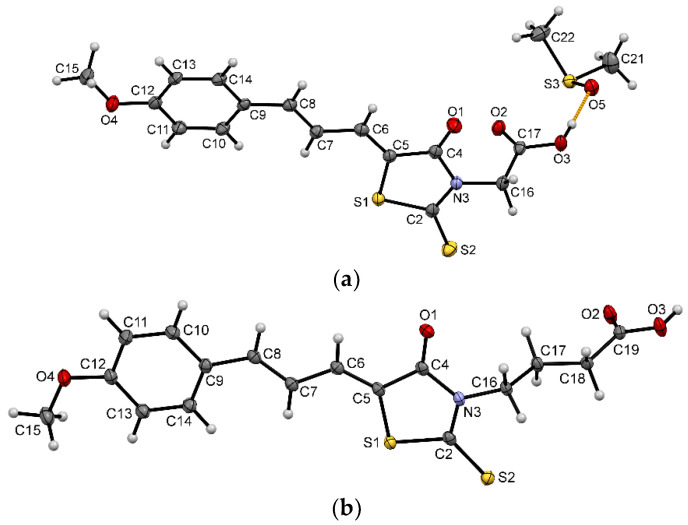
The molecular geometries of (**a**) **2** and (**b**) **4**, with the atom-numbering schemes. Displacement ellipsoids are drawn at the 50% probability level. The intermolecular hydrogen bond between **2** and DMSO is depicted as an orange dashed line.

For two representative compounds, active **2** and inactive **4**, X-ray crystallographic analysis was carried out. The projections of molecular geometries in the crystals of **2** and **4** are presented in Figure 2. The asymmetric unit of **2** consists of one molecule of the investigated compound and one solvent molecule of dimethyl sulfoxide (DMSO), while **4** crystallizes only with one molecule in the asymmetric unit.

The investigated compounds possess two double bonds C5=C6 and C7=C8. As shown in Figure 2 for both presented compounds the same isomer (5Z, 7E) in crystals is observed. This isomer was also observed in other crystal structures determined earlier, containing rhodanine and aromatic rings linked with the same chain construct [10,29]. 

The 5-cinnamylidenerhodanine fragment is conjugated and almost planar. The values of single bonds (C6–C7 and C8–C9) in the linker are between the values of single and double bonds, namely 1.431(2) Å and 1.459(2) Å for **2**, and 1.433(2) Å and 1.455(2) Å for **4**. The angle between the planes containing rhodanine and aromatic rings is 11.71(8)° and 0.32(9)°, for **2** and **4**, respectively. Similar values of bond lengths and interplanar angles are in agreement with these observed in the crystal structures of other derivatives containing this fragment [10,29].

**Figure 3:**
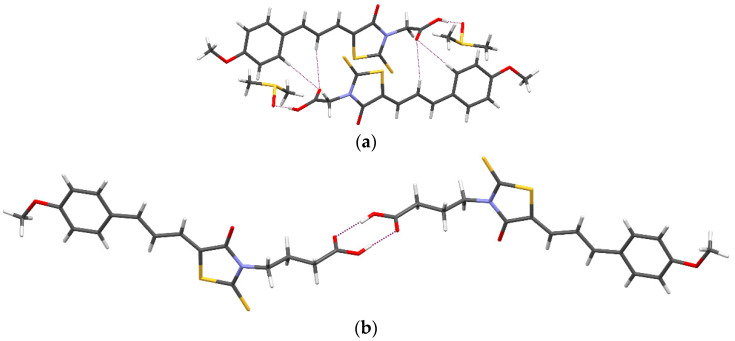
Intermolecular interactions of two molecules in the crystals of (**a**) **2** and (**b**) **4**. Dashed purple lines indicate the formed hydrogen bonds.

The main intermolecular interactions motif is related to the O-H…O hydrogen bonds formation. One molecule of **2** interacts by carboxyl group with one molecule of DMSO (Figure 3a), while in the case of **4,** two molecules related by the inversion centre interact by carboxyl groups (Figure 3b). Furthermore, the crystal structures are stabilized by C-H…O and C-H…S contacts.

#### 2.2.2. Anthelmintic Activity and Molecular Geometry

**Figure 4:**
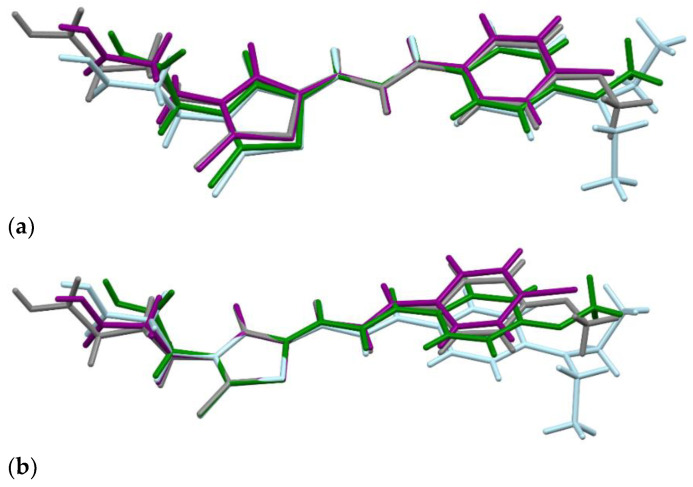
The overlap of (**a**) the linkers between two rings and (**b**) rhodanine rings of **2** (green), **4** (grey), **14** (purple) and **16** (light blue).

We have analyzed the geometries of active compound **2** and inactive **4** in the crystals and compared them with two other derivatives, namely **14** and **16**, for which the crystal structures were determined earlier [10]. The compared compounds differ in the length of the linker between the rhodanine ring and carboxyl group and in the substituent at the aromatic ring. Figure 4 presents the overlap of all four compared molecules. The analyzed compounds show small differences in planarity of the 5-cinnamylidenerhodanine moiety (Figure 4a). The biologically active compound **2** shows the biggest deviation from the planarity of this fragment in comparison to the other three non-active compounds. Furthermore, the carbon atom of the methoxy substituent of **2** points to the opposite direction in comparison to **4**. Mutual orientation of carboxyl group and rhodanine ring also shows differences (Figure 4b). 

### 2.3. Anthelmintic Activity

**Figure 5:**
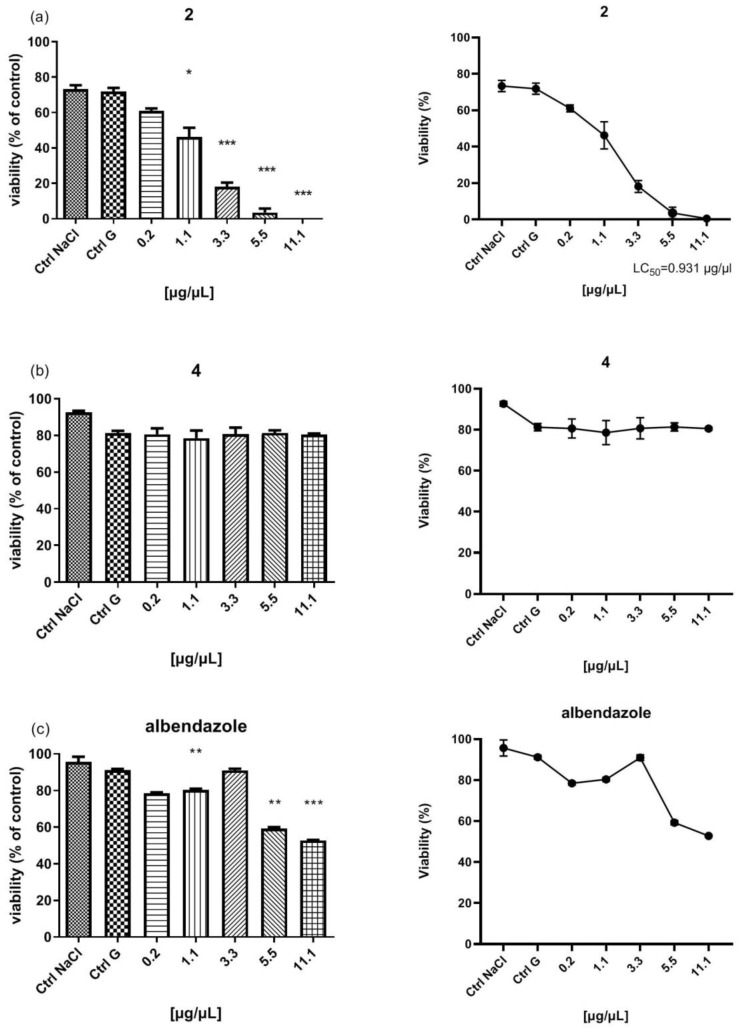
Viability (%) of nematodes of the genus *Rhabditis* sp. after 24-h exposure to increasing concentrations of (**a**) **2**, (**b**) **4** and (**c**) albendazole; (*p* < 0.05 *, *p* < 0.01 **, *p* < 0.001 ***). The test was carried out in three replications.

**Figure 6:**
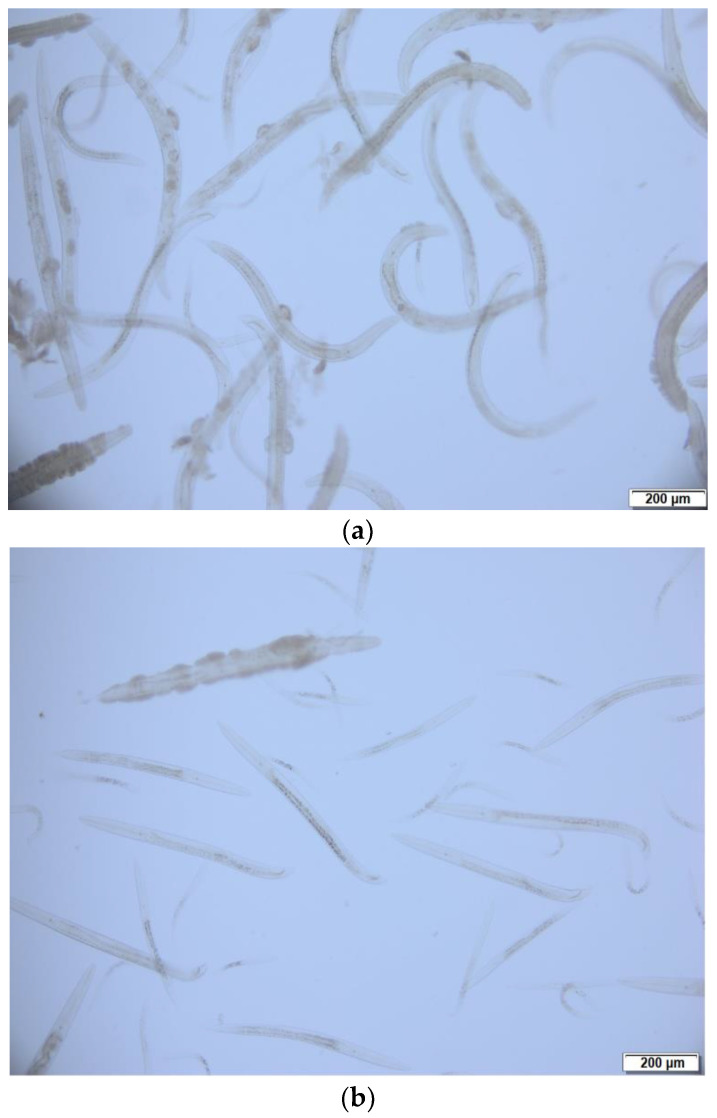
The nematode culture of *Rhabditis* sp. (**a**) Control 0.6% NaCl—alive, very mobile, (**b**) after application of **2**—dead, non-mobile.

Sixteen cinnamylidene derivatives of rhodanine were evaluated against anthelmintic activity. Among newly synthesized methoxy derivatives (**1**–**11**), only **2** shows bioactivity against *Rhabditis* sp. For the active compound, the survival rate of 50% (lethal concentration 50) LC_50_ = 0.931 µg/µL was determined (Figure 5a). The microscopic image showed dead and immobile nematodes (Figure 6). The other methoxy derivatives have no anthelmintic activity (Figure 5b). In Figure 5c is also presented the anthelmintic activity of albendazole, the drug used as a positive control in performed experiments (LC_50_ = 19.24 µg/µL). Amongst chloro, nitro and diethylamine derivatives (**12**–**16**), none of the compounds exhibited anthelmintic activity.

### 2.4. Studies on Potential Mechanism of Action

#### 2.4.1. Docking Studies

For a deeper insight of the observed anthelmintic activity of **2** compared to the inactive **4** and two other inactive derivatives **14** and **16** with known crystal structures [10] the molecular docking study was performed. In analogy to albendazole (a chosen control probe in this study), we set the hypothesis that the mechanism of action of **2** is through the inhibition of microtubule polymerisation. Thus, the colchicine highly hydrophobic binding pocket postulated as the albendazole putative binding site [30], was selected for the molecular docking experiment.

**Figure 7:**
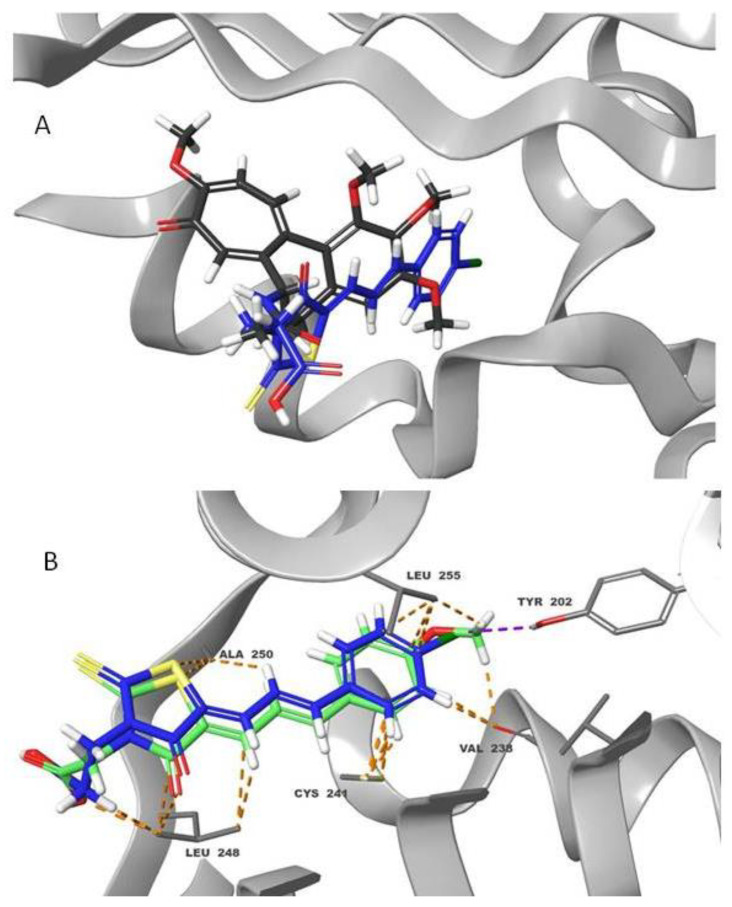
Representation of the docked poses of the investigated ligands in the tubulin β-2B binding site. (**A**)—docked colchicine (black carbon atoms) and compound **14** (blue carbon atoms); the rhodanine derivative penetrates deeper the protein binding pocket. (**B**)—superposition of the top-ranked poses of active **2** (light green carbon atoms) and inactive **14** (blue carbon atoms) showing the corresponding interactions within the binding site.

The re-docking experiment performed for the pre-prepared tubulin structure in complex with colchicine (PDB ID: 5NM5) [31] confirmed the good performance of the designed methodology, resulting in 0.77 RMSD calculated for corresponding heavy atoms of the docked colchicine and the active conformer observed in the crystal structure (PDB ID: 5NM5). The native ligand reached the best docking score (Table 2). Surprisingly, the second-best fit was obtained for inactive **14**, followed by active **2**. Both mentioned ligands adopt nearly identical poses in the tubulin β-2B hydrophobic pocket, allowing deeper penetration of the molecule in the binding site in comparison to the colchicine original location (Figure 7). The best-ranked rhodanine derivatives **2** and **14** form hydrophobic interactions with Cys241, Leu248, Ala250, Leu 255, and weak hydrogen bonds with Val238 and Tyr202. The last residue is not accessible for the colchicine molecule due to the rigidity and less elongated shape of the alkaloid derivative compared to rhodanine derivatives. Only slightly less prominent in the predicted binding is **4**. The longer linker between rhodanine and carboxyl group increases the flexibility of the molecule with no additional interactions observed.

Despite the elongated shape of the albendazole molecule, it does not penetrate the binding site as deep as the rhodanine derivatives, which could explain its lower position in the ranking list. Interestingly, the molecule of **16** adopts an opposite orientation compared to other studied rhodanine derivatives. The lowest position in the ranking and observed different pose may be related to the relative flexibility of the diethylamine substituent, which can cause steric hindrance in the deeper regions of the tubulin β-2B binding pocket. With this distinct orientation of the molecule, the carboxylic functional group interacts with Tyr 202, forming a strong O-H…O hydrogen bond, which does not tip the balance in favour of this pose.

#### 2.4.2. Prediction of Selected Physicochemical Properties

The above-described docking results suggest higher affinities of compounds **2**, **4**, and **14** towards postulated target protein, tubulin β-2B, compared to albendazole. However, it still does not explain why only **2** exhibits the anthelmintic activity in the biological test. To find the answer to the observed results, we decided to estimate selected physicochemical properties, which can shed some light on the obtained experimental results. We examined predicted measures for solubility (log S) and lipophilicity (log P). The obtained results (Table 3, data for colchicine are shown only for comparison) suggest only moderate solubility with albendazole being the leader amongst the studied compounds.

An interesting relation can be spotted for log P predicted values. The active compound **2** has the lowest value of log P for the studied compounds, suggesting the least lipophilic properties of this molecule. As it was suggested by Kerboeuf and Guégnard [32], anthelmintics can act as substrates or modulators of the P-glycoprotein (P-gp) activity in the nematode. This ABC-transporter protein is responsible for drug resistance, including observed lowering anthelmintic activity in mammals [33]. P-gp recognizes its substrates in the inner region of the cell membrane and transports lipophilic or amphiphilic molecules [34]. Thus, modification of molecular properties (especially the change in lipophilicity level) by inserting different substituents for a series of bioactive compounds can modulate the compound’s impact on P-gp activity, which can be called a “log P-response” relationship [35]. For this reason, the stronger anthelmintic activity observed for **2** in comparison to albendazole could be explained by the lower log P value and the related increased bioavailability. This can also suggest that the higher lipophilicity for the remaining rhodanine derivatives (**4**, **14** and **16**) may be the limiting factor forbidding the molecules to reach the target protein in vivo.

## 3. Materials and Methods

### 3.1. Chemical Synthesis

Reagents used for synthesis were purchased from Sigma-Aldrich (Munich, Germany) and Across Organics and were used without further purification. 

The melting points were determined on a Boetius apparatus (Jena, Germany) and are uncorrected.

An AmaZon EDT apparatus (Bremen, Germany) equipped with an electrospray ion source and an ion trap analyzer was used to perform the mass spectrometry analysis. The samples were introduced into the apparatus after dissolving them in a mixture of methanol and chloroform (1: 1 *v/v*) with the addition of 0.1% vol of formic acid. The scanning was carried out in the range of 100–700 *m/z*, accumulating approximately 300,000 ions in one cycle. Positive or negative ion polarization was used, depending on the properties of the tested sample.

The IR spectra were recorded on a JASCO FT IR-670 Plus instrument in KBr pellets (1 mg sample/400 mg KBr).

The ^1^H-NMR spectra and ^13^C-NMR spectra were recorded on a JEOL JNM-ECZR500 RS1 (ECZR version) at 500 and 126 MHz, respectively, and were reported in parts per million (ppm) using deuterated solvent for calibration (DMSO-d6). The coupling constants values (*J*) were reported in hertz (Hz), and the splitting patterns were designated as follows: br s. (broad singlet), br d. (broad doublet), s (singlet), d (doublet), t (triplet), q (quartet), dd (doublet of doublets), m (multiplet).

#### General Procedures for the Synthesis of Final Compounds **1**–**11**

The appropriate rhodanine or rhodanine-3-alkanoic acid (5.0 mmol), molecular sieves 4A (5 g), isopropyl alcohol (30 cm^3^), appropriate aldehyde (5.5 mmol) and triethylamine (25.0 mmol) were placed into a flask. The mixture was heated under a reflux condenser for 4–5 h in argon. Upon completion of the reaction, the hot solution was immediately filtered. The filtrate was cooled and 2 M hydrochloric acid solution was added in order to obtain an acidic solution. Next, the obtained precipitate was filtered using a Büchner funnel and crystallized from acetic anhydride or glacial acetic acid.

##### 5-(4′-Methoxycinnamylidene)-rhodanine (**1**)

m.p. 235–237 °C, MS [M − 1]^−^ 276, IR (1 mg/400 mg KBr) cm^−1^: 3219.6 N-H, 1704.8 C=O conj., 1640.2 C=C exo., 1578.5 C=CH-CH=CH-Ar, 1463.7 CH_3_, 1314.3 C-O-C, 1152.3 C=S, ^1^H NMR (500 MHz, DMSO-d_6_), δ: 13.52 (s, 1H, H-N), 7.61 (d, *J*= 8.9 Hz, 2H, HC2 Ar, HC6 Ar), 7.30 (d, *J* = 10.9 Hz, 1H, C=CH-CH=CH-Ar), 7.25 (d, *J* = 15.2 Hz, 1H, C=CH-CH=CH-Ar), 6.94 (d, *J*= 8.9 Hz, 2H, HC3 Ar, HC5 Ar), 6.82 (dd, *J* = 15.0 Hz, *J* = 11.6 Hz, 1H, C=CH-CH=CH-Ar), 3.77 (s, 3H, OCH_3_), ^13^C NMR (126 MHz, DMSO-d_6_), δ: 195.7 (S=C-S), 169.2 (N-C=O), 161.5 (C4 Ar), 145.5 (C=CH-CH=CH-Ar), 133.3 (C=CH-CH=CH-Ar),130.4 (C=CH-CH=CH-Ar), 128.9 (C=CH-CH=CH-Ar), 125.8 (C2 Ar), 115.2 (C3 Ar), 55.9 (OCH_3_).

##### 5-(4′-Methoxycinnamylidene)-rhodanine-3-etanoic Acid (**2**)

m.p. 268–270 °C, MS [M + 1]^+^ 336, IR (1 mg/400 mg KBr) cm^−1^: 1715.4C=O, 1697.1 C=O conj., 1641.2 C=C exo., 1574,6 C=CH-CH=CH-Ar, 1464.7 CH_3_, 1394.3 CH_2_, 1310.4 C-O-C, 1163.8 C=S, ^1^H NMR (500 MHz, DMSO-d_6_), δ: 13.32 (s, 1H, HOOC-), 7.66 (d, *J* = 8.9 Hz, 2H, HC2 Ar, HC6 Ar), 7.55 (d, *J* = 10.9 Hz, 1H, C=CH-CH=CH-Ar), 7.36 (d, *J* = 14.9 Hz, 1H, C=CH-CH=CH-Ar), 6.98 (m, 3H, C=CH-CH=CH-Ar, HC3 Ar, HC5 Ar), 4.66 (s, 2H, HOOCH_2_N), 3.78 (s, 3H, OCH_3_), ^13^C NMR (126 MHz, DMSO-d_6_), δ: 193.3 (S=C-S), 167.9 (HOOC-), 166.2 (N-C=O), 161.7 C4 Ar), 146.9 (C=CH-CH=CH-Ar), 135.7 (C=CH-CH=CH-Ar), 128.8 (C=CH-CH=CH-Ar), 121.8 (C=CH-CH=CH-Ar), 121.8 (C1 Ar), 115.1 (C3 Ar), 56.0 (OCH_3_), 45.4 (HOOCCH_2_).

##### 5-(4′-Methoxycinnamylidene)-rhodanine-3-propanoic Acid (**3**)

m.p. 244–246 °C, MS [M − 1]^−^ 348, IR (1 mg/400 mg KBr) cm^−1^: 1705.7 C=O, 1684.5 C=O conj., 1641.3 C=C exo., 1574.6 C=CH-CH=CH-Ar, 1463.7 CH_3_, 1379.8 CH_2_, 1310.4 C-O-C, 1160.0 C=S, ^1^H NMR (500 MHz, DMSO-d_6_), δ: 12.43 (s, 1H, HOOC), 7.64 (d, *J* = 8.9 Hz, 2H, HC2 Ar, HC6 Ar), 7,48 (d, *J* = 11.7 Hz, 1H, C=CH-CH=CH-Ar), 7.32 (d, *J* = 15.2 Hz, 1H, C=CH-CH=CH-Ar), 6.95 (d, *J* = 8.9 Hz, 2H, HC3 Ar, HC5 Ar), 6.91 (dd, *J* = 15.2 Hz, *J* = 11.7 Hz, 1H, C=CH-CH=CH-Ar), 4.15 (t, *J* = 7.9 Hz, 2H, HOOCCH_2_CH_2_N), 3.77 (s, 3H, OCH_3_), 2.57 (t, *J* = 7.7 Hz, 2H, HOOCCH_2_CH_2_N), ^13^C NMR (126 MHz, DMSO-d_6_), δ: 193.3 (S=C-S), 172.3 (HOOC), 166.6 (N-C=O, 161.6 (C4 Ar), 146.2 (C=CH-CH=CH-Ar), 134.8 (C=CH-CH=CH-Ar), 130.6 (C2 Ar), 128.8 (C1 Ar), 122.4 (C=CH-CH=CH-Ar), 121.9 (C=CH-CH=CH-Ar), 115.3 (C3 Ar), 55.9 (OCH_3_), 40.6 (HOOCCH_2_CH_2_), 31.4 (HOOCCH_2_CH_2_).

##### 5-(4′-Methoxycinnamylidene)-rhodanine-3-butanoic Acid (**4**)

m.p. 208–211 °C, MS [M + 1]^+^ 364, IR (1 mg/400 mg KBr) cm^−1^: 1694.1 C=O, 1684.5 C=O conj., 1642.1 C=C exo., 1574.6 C=CH-CH=CH-Ar, 1453.1 CH_3_, 1390.4 CH_2_, 1303.6 C-O-C, 1157.1 C=S, ^1^H NMR (500 MHz, DMSO-d_6_), δ: 12.07 (s, 1H, HOOC), 7.63 (d, *J* = 8.9 Hz, 2H, HC2 Ar, HC6 Ar), 7.45 (d, *J* = 10.9 Hz, 1H, C=CH-CH=CH-Ar), 7.30 (d, *J* = 14.9 Hz, 1H, C=CH-CH=CH-Ar), 6.95 (d, *J* = 8.9 Hz, 2H, HC3 Ar, HC5 Ar), 6.89 (dd, *J* = 15.0 Hz, *J* = 11.6 Hz, 1H, C=CH-CH=CH-Ar), 3.99 (t, *J* = 7.0 Hz, 2H, HOOCCH_2_CH_2_CH_2_), 3.77 (s, 3H, OCH_3_), 2.24 (t, *J* = 7.3 Hz, 2H, HOOCCH_2_CH_2_CH_2_), 1.85–1.80 (m, 2H, HOOCCH_2_CH_2_CH_2_), ^13^C NMR (126 MHz, DMSO-d_6_), δ: 193.7 (S=C-S), 174.2 (HOOC), 167.0 (N-C=O), 161.6 (C4 Ar), 146.0 (C=CH-CH=CH-Ar), 134.6 (C=CH-CH=CH-Ar), 130.6 (C2 Ar), 128.9 (C1 Ar), 122.6 (C=CH-CH=CH-Ar), 121.9 (C=CH-CH=CH-Ar), 115.1 (C3 Ar), 55.9 (OCH_3_), 44.1 (HOOCH_2_CH_2_CH_2_), 31.5 (HOOCCH_2_CH_2_CH_2_), 22.6 (HOOCCH_2_CH_2_CH_2_).

##### 5-(4′-Methoxycinnamylidene)-rhodanine-3-pentanoic Acid (**5**)

m.p. 211–213 °C, MS [M + 1]^+^ 378, IR (1 mg/400 mg KBr) cm^−1^: 1699.0 C=O, 1684.5 C=O conj., 1641.8 C=C exo., 1573.0 C=CH-CH=CH-Ar, 1460.8 CH_3_, 1388.5 CH_2_, 1312.2 C-O-C, 1155.2 C=S, ^1^H NMR (500 MHz, DMSO-d_6_), δ: 11.98 (s, 1H, HOOC), 7.63 (d, *J* = 8.9 Hz, 2H, HC2 Ar, HC6 Ar), 7.46 (d, *J* = 8.9 Hz, 1H, C=CH-CH=CH-Ar), 7.30 (d, *J* = 15.2 Hz, 1H, C=CH-CH=CH-Ar), 6.95 (d, *J* = 8.9 Hz, 2H, HC3 Ar, HC5 Ar), 6.89 (dd, *J* = 15.0 Hz, *J* = 11.6 Hz, 1H, C=CH-CH=CH-Ar), 3.94 (t, *J* = 7.3 Hz, 2H, HOOCH_2_CH_2_CH_2_CH_2_), 3.77 (s, 3H, OCH_3_), 2.21 (t, *J* = 7.3 Hz, 2H, HOOCH_2_CH_2_CH_2_CH_2_), 1.63–1.57 (m, 2H, HOOCH_2_CH_2_CH_2_CH_2_), 1.49–1.43 (m, 2H, HOOCH_2_CH_2_CH_2_CH_2_), ^13^C NMR (126 MHz, DMSO-d_6_), δ: 193.4 (S=C-S), 174.6 (HOOC), 166.8 (N-C=O), 161.6 (C4 Ar), 146.2 (C=CH-CH=CH-Ar), 134.7 (C=CH-CH=CH-Ar), 130.6 (C2 Ar), 128.9 (C1 Ar), 122.4 (C=CH-CH=CH-Ar), 121.9 (C=CH-CH=CH-Ar), 115.1 (C3 Ar), 55.9 (OCH_3_), 44.3 (HOOCH_2_CH_2_CH_2_CH_2_), 33.6 (HOOCCH_2_CH_2_CH_2_CH_2_), 26.5 (HOOCCH_2_CH_2_CH_2_CH_2_), 22.6 (HOOCCH_2_CH_2_CH_2_CH_2_).

##### 5-(4′-Methoxycinnamylidene)-rhodanine-3-hexanoic Acid (**6**)

m.p. 174–176 °C, MS [M + 1]^+^ 392, IR (1 mg/400 mg KBr) cm^−1^: 1707.7 C=O, 1685.5 C=O conj., 1640.1 C=C exo., 1578.5 C=CH-CH=CH-Ar, 1464.8 CH_3_, 1385.5 CH_2_, 1307.5 C-O-C, 1153.2 C=S, ^1^H NMR (500 MHz, DMSO-d_6_), δ: 11.95 (s, 1H, HOOC), 7.63 (d, *J* = 8.9 Hz, 2H, HC2 Ar, HC6 Ar), 7.43 (d, *J* = 11.5 Hz, 1H, C=CH-CH=CH-Ar), 7.26 (d, *J* = 15.2 Hz, 1H, C=CH-CH=CH-Ar), 6.93 (d, *J* = 8.9 Hz, 2H, HC3 Ar, HC5 Ar), 6.85 (dd, *J* = 15.0 Hz, *J* = 11.6 Hz, 1H, C=CH-CH=CH-Ar), 3.94 (t, *J* = 7.4 Hz, 2H, HOOCCH_2_CH_2_CH_2_CH_2_CH_2_-), 3.76 (s, 3H, OCH_3_), 2.15 (t, *J* = 7.3 Hz, 2H, HOOCCH_2_CH_2_CH_2_CH_2_CH_2_-), 1.57 (q, *J* = 7.4 Hz, 2H, HOOCCH_2_CH_2_CH_2_CH_2_CH_2_-), 1.46 (q, 2H, HOOCCH_2_CH_2_CH_2_CH_2_CH_2_-), 1.25 (q, 2H, HOOCCH_2_CH_2_CH_2_CH_2_CH_2_-), ^13^C NMR (126 MHz, DMSO-d_6_), δ: 193.4 (S=C-S), 174.9 (HOOC), 166.7 (N-C=O), 161.6 (C4 Ar), 146.1 (C=CH-CH=CH-Ar), 134.7 (C=CH-CH=CH-Ar), 130.6 (C2 Ar), 128.8 (C1 Ar), 122.4 (C=CH-CH=CH-Ar), 121.8 (C=CH-CH=CH-Ar), 115.0 (C3 Ar), 55.9 (OCH_3_), 44.4 (HOOCCH_2_CH_2_CH_2_CH_2_CH_2_-), 33.9 (HOOCCH_2_CH_2_CH_2_CH_2_CH_2_-), 26.6 (HOOCCH_2_CH_2_CH_2_CH_2_CH_2_-), 26.2 (HOOCCH_2_CH_2_CH_2_CH_2_CH_2_-), 24.6 (HOOCCH_2_CH_2_CH_2_CH_2_CH_2_-).

##### 5-(4′-Methoxycinnamylidene)-rhodanine-3-undecanoic Acid (**7**)

m.p. 153–155 °C, MS [M − 1]^−^ 460, IR (1 mg/400 mg KBr) cm^−1^: 1711.5 C=O, 1698.0 C=O conj., 1640.2 C=C exo., 1578.5 C=CH-CH=CH-Ar, 1466.6 CH_3_, 1385.6 CH_2_, 1321.0 C-O-C, 1160.9 C=S, ^1^H NMR (500 MHz, DMSO-d_6_), δ: 11.90 (s, 1H, HOOC), 7.62 (d, *J* = 8.9 Hz, 2H, HC2 Ar, HC6 Ar), 7.45 (d, *J* = 10.9 Hz, 1H, C=CH-CH=CH-Ar), 7.29 (d, *J* = 15.2 Hz, 1H, C=CH-CH=CH-Ar), 6.94 (d, *J* = 8.9 Hz, 2H, HC3 Ar, HC5 Ar), 6.88 (dd, *J* = 15.0 Hz, *J* = 11.6 Hz, 1H, C=CH-CH=CH-Ar), 3.92 (t, *J* = 7.4 Hz, 2H, HOOC-CH_2_CH_2_CH_2_CH_2_CH_2_CH_2_CH_2_CH_2_CH_2_CH_2_-), 3.76 (s, 3H, OCH_3_), 2.13 (t, *J* = 7.3 Hz, 2H, HOOC-CH_2_CH_2_CH_2_CH_2_CH_2_CH_2_CH_2_CH_2_CH_2_CH_2_-), 1.55 (d, *J* = 14.0 Hz, 2H, HOOC-CH_2_CH_2_CH_2_CH_2_CH_2_CH_2_CH_2_CH_2_CH_2_CH_2_-), 1.43 (d, *J* = 14.3, 2H, HOOC-CH_2_CH_2_CH_2_CH_2_CH_2_CH_2_CH_2_CH_2_CH_2_CH_2_-), 1,20 (d, *J* = 17.8 Hz, 12H, HOOC-CH_2_CH_2_CH_2_CH_2_CH_2_CH_2_CH_2_CH_2_CH_2_CH_2_-), ^13^C NMR (126 MHz, DMSO-d_6_), δ: 193.3 (S=C-S), 175.0 (HOOC), 166.8 (N-C=O), 161.6 (C4 Ar), 146.1 (C=CH-CH=CH-Ar), 134.7 (C=CH-CH=CH-Ar), 128.8 (C=CH-CH=CH-Ar), 122.4 (C=CH-CH=CH-Ar), 121.9 (C1 Ar), 115.1 (C3 Ar), 55.9 (OCH_3_), 44.6 (HOOC-CH_2_CH_2_CH_2_CH_2_CH_2_CH_2_CH_2_CH_2_CH_2_CH_2_-), 34.2 (HOOC-CH_2_CH_2_CH_2_CH_2_CH_2_CH_2_CH_2_CH_2_CH_2_CH_2_-), 29.3 (HOOC-CH_2_CH_2_CH_2_CH_2_CH_2_CH_2_CH_2_CH_2_CH_2_CH_2_-), 29.3 (HOOC-CH_2_CH_2_CH_2_CH_2_CH_2_CH_2_CH_2_CH_2_CH_2_CH_2_-), 29.3 (HOOC-CH_2_CH_2_CH_2_CH_2_CH_2_CH_2_CH_2_CH_2_CH_2_CH_2_-), 29.1 (HOOC-CH_2_CH_2_CH_2_CH_2_CH_2_CH_2_CH_2_CH_2_CH_2_CH_2_-), 29.0 (HOOC-CH_2_CH_2_CH_2_CH_2_CH_2_CH_2_CH_2_CH_2_CH_2_CH_2_-), 26.8 (HOOC-CH_2_CH_2_CH_2_CH_2_CH_2_CH_2_CH_2_CH_2_CH_2_CH_2_-), 26.7 (HOOC-CH_2_CH_2_CH_2_CH_2_CH_2_CH_2_CH_2_CH_2_CH_2_CH_2_-), 25.0 (HOOC-CH_2_CH_2_CH_2_CH_2_CH_2_CH_2_CH_2_CH_2_CH_2_CH_2_-).

##### 5-(2′-Methoxycinnamylidene)-rhodanine (**8**)

m.p. 210–211 °C, MS [M − 1]^−^ 276, IR (1 mg/400 mg KBr) cm^−1^: 3149.1 N-H, 1709.6 C=O conj., 1641.1 C=C exo., 1597.3 C=CH-CH=CH-Ar, 1467.6 CH_3_, 1300.8 C-O-C, 1158.0 C=S, ^1^H NMR (500 MHz, DMSO-d_6_), δ: 13.57 (s, 1H, HN-), 7.71 (dd, *J* = 7.7 Hz, *J* = 1.4 Hz, 1H, HC6 Ar), 7.48 (d, *J* = 15.2 Hz, 1H, C=CH-CH=CH-Ar), 7.37–7.33 (m, 2H, C=CH-CH=CH-Ar), 7.04 (d, *J* = 8.0 Hz, 1H, HC3 Ar), 6.97–6.91 (m, 2H, HC4 Ar, HC5 Ar), 3.83 (s, 3H, OCH_3_), ^13^C NMR (126 MHz, DMSO-d_6_), δ: 195.6 (S=C-S), 169.1 (N-C=O), 158.3 (C-OCH_3_), 141.4 (C=CH-CH=CH-Ar), 137.4 (C=CH-CH=CH-C), 133.4 (C=CH-CH=CH-Ar), 132.4 (C4 Ar), 128.9 (C5 Ar), 126.9 (C=CH-CH=CH-Ar),124.6 (C=CH-CH=CH-Ar), 121.3 (C4 Ar), 117.0 (C4 Ar), 112.3 (C4 Ar), 56.2 (O-CH_3_).

##### 5-(2′-Methoxycinnamylidene)-rhodanine-3-etanoic Acid (**9**)

m.p. 289–290 °C, MS [M + 1]^+^ 336, IR (1 mg/400 mg KBr) cm^−1^: 1725.0 C=O, 1700.9 C=O conj., 1641.3 C=C exo., 1581.3 C=CH-CH=CH-Ar, 1466.6 CH_3_, 1405.8 CH_2_, 1325.8 C-O-C, 1159.9 C=S, ^1^H NMR (500 MHz, DMSO-d_6_), δ: 13.39 (s, 1H, HOOC-),7.75 (dd, *J* = 7.9 Hz, *J* = 1.6 Hz, 1H, HC6 Ar), 7.62–7.57 (m, 2H, C=CH-CH=CH-Ar), 7.38–7.35 (m, 1H, HC4 Ar), 7.08–7.00 (m, 2H, HC3 Ar, HC5 Ar), 6.94 (t, *J*= 7.4 Hz, 1H, C=CH-CH=CH-Ar), 4.65 (s, 2H, HOOC-CH_2_-N), 3.84 (d, *J* = 7.2 Hz, 3H, OCH_3_), ^13^C NMR (126 MHz, DMSO-d_6_), δ: 193.2 (S=C-S), 167.9 (HOOC-), 166.1 (N-C=O), 158.1 (C-OCH_3_), 140.1 (C=CH-CH=CH-Ar), 135.8 (C=CH-CH=CH-Ar), 129.0 (C=CH-CH=CH-Ar), 124.4 (C=CH-CH=CH-Ar), 124.3 (C=CH-CH=CH-Ar), 122.9 (C6 Ar), 121.3 (C5 Ar), 112.3 (C3 Ar), 56.2 (O-CH_3_), 45.4 (HOOCCH_2_-).

##### 5-(3′-Methoxycinnamylidene)-rhodanine (**10**)

m.p. 253–254 °C, MS [M − 1]^−^ 276, IR (1 mg/400 mg KBr) cm^−1^: 3160.8 N-H, 1693.2 C=O conj., 1641.1 C=C exo., 1577.5 C=CH-CH=CH-Ar, 1465.6 CH_3_, 1304.6 C-O-C, 1161.9 C=S, ^1^H NMR (500 MHz, DMSO-d_6_), δ: 13.60 (s, 1H, HN-), 7.31–7.24 (m, 3H, C=CH-CH=CH-Ar), 7.23 (s, 1H, HC2 Ar), 7.19 (d, *J* = 7.7 Hz, 1H, HC6 Ar), 7.00 (dd, *J* = 15.2 Hz, *J* = 11.5 Hz, 1H, HC5 Ar), 6.92 (dd, *J* = 8.0 Hz, *J* = 2.0 Hz, 1H, HC4 Ar), 3.76 (s, 3H, OCH_3_), ^13^C NMR (126 MHz, DMSO-d_6_), δ: 195.9 (S=C-S), 169.3 (N-C=O), 160.2 (C3 Ar), 145.2 (C=CH-CH=CH-Ar), 137.5 (C1 Ar), 130.5 (C5 Ar), 127.7 (C=CH-CH=CH-Ar), 124.6 (C=CH-CH=CH-Ar), 121.5 (C6 Ar), 116.6 (C4 Ar), 112.9 (C2 Ar), 55.8 (O-CH_3_).

##### 5-(3′-Methoxycinnamylidene)-rhodanine-3-etanoic Acid (**11**)

m.p. 234–235 °C, MS [M + 1]^+^ 336, IR (1 mg/400 mg KBr) cm^−1^: 1718.3 C=O, 1709.6 C=O conj., 1641.1 C=C exo., 1575.6 C=CH-CH=CH-Ar, 1466.6 CH_3_, 1398.1 CH_2_, 1326.8 C-O-C, 1167.7 C=S, ^1^H NMR (500 MHz, DMSO-d_6_), δ: 13.39 (s, 1H, HOOC-), 7.55 (d, *J* = 11.5 Hz, 1H, C=CH-CH=CH-Ar), 7.35 (d, *J* = 15.2 Hz, 1H, C=CH-CH=CH-Ar), 7.32–7.26 (m, 2H, HC5 Ar, HC6 Ar), 7.22 (d, *J* = 7.4 Hz, 1H, HC2 Ar), 7.15–7.09 (m, 1H, C=CH-CH=CH-Ar), 6.94 (d, *J* = 8.3 Hz, 1H, HC4 Ar), 4.66 (s, 2H, HOOC-CH_2_-N), 3.77 (s, 3H, OCH_3_), ^13^C NMR (126 MHz, DMSO-d_6_), δ: 193.5 (S=C-S), 167.9 (HOOC-), 166.3 (N-C=O), 160.2 (C-OCH_3_), 146.5 (C=CH-CH=CH-Ar), 137.4 (C=CH-CH=CH-C), 134.9 (C=CH-CH=CH-Ar), 130.5 (C5 Ar), 124.4 (C=CH-CH=CH-Ar),123.7 (C=CH-CH=CH-Ar), 121.8 (C6 Ar), 117.0 (C4 Ar), 113.0 (C2 Ar), 55.8 (O-CH_3_), 45.4 (HOOC-CH_2_-N).

### 3.2. X-ray Crystallographic Studies

Crystals suitable for an X-ray structure analysis were obtained by slow evaporation of the solvent at ambient conditions from a mixture of dimethyl sulfoxide and acetonitrile for **2** and from ethanol for **4**.

Data for the single crystal of **2** were collected using the Oxford Diffraction SuperNova four circle diffractometer, equipped with the MoKα (0.71073 Å) radiation source and graphite monochromator, while for **4** were collected using the XtaLAB Synergy-S diffractometer, equipped with the CuKα (1.54184 Å) radiation source and graphite monochromator. Positions of all non-hydrogen atoms were determined by direct methods using the SIR-2014 [36] program. Refinement and further calculations were carried out using the SHELXL-2018 program [37]. All non-hydrogen atoms were refined anisotropically using weighted full-matrix least-squares on F^2^. The hydrogen atoms bonded to carbons were included in the structure at idealized positions and were refined using a riding model. Hydrogen atoms attached to oxygen atoms were found from the difference Fourier map and refined without restraints. For molecular graphics the MERCURY [38] program was used.

**2**: C_15_H_13_NO_4_S_2_·C_2_H_6_OS M_r_ = 413.51, crystal size = 0.05 × 0.17 × 0.92 mm^3^, triclinic, space group P$\bar{1}$, a = 7.9242(3) Å, b = 8.0696(3) Å, c = 15.7132(6) Å, α = 89.059(3)°, β = 89.935(3)°, γ = 70.006(3)°, V = 944.08(6) Å^3^, Z = 2, T = 130(2) K, 12823 reflections collected, 4415 unique reflections (R_int_ = 0.0278), R1 = 0.0336, wR2 = 0.0777 [I > 2σ(I)] and R1 = 0.0457, wR2 = 0.0852 [all data].

**4**: C_17_H_17_NO_4_S_2_, M_r_ = 363.43, crystal size = 0.02 × 0.06 × 0.67 mm^3^, monoclinic, space group P2_1_/c, a = 17.3826(1) Å, b = 4.7425(1) Å, c = 20.2816(2) Å, β = 94.305(1)°, V = 1667.24(4) Å^3^, Z = 4, T = 100(2) K, 44217 reflections collected, 3397 unique reflections (R_int_ = 0.0637), R1 = 0.0267, wR2 = 0.0700 [I > 2σ(I)] and R1 = 0.0281, wR2 = 0.0713 [all data].

CCDC 2154601-2154602 contain the supplementary crystallographic data. These data can be obtained free of charge from The Cambridge Crystallographic Data Centre via www.ccdc.cam.ac.uk/data_request/cif (accessed on 24 February 2022).

### 3.3. Anthelmintic Activity Assay

Nematodes of the genus *Rhabditis* sp. were used for the anthelmintic activity experiments. The nematodes were cultured following the procedure developed by A. Bogucka-Kocka and P. Kołodziej at the Department of Biology and Genetics of the Medical University of Lublin [39]. Test derivatives were suspended in 60% glycerol and sonicated using an ultrasonic homogenizer (Hielscher Ultrasound Technology, Teltow, Germany). The initial homogeneous emulsion was prepared at a concentration of 100 µg/µL. The experiment was performed on 24-well plates. The test compounds were added to nematode cultures at the following concentrations: 0.2 µg/µL; 1.1 µg/µL; 3.3 µg/µL; 5.5 µg/µL; 11.1 µg/µL. Additionally, separate cultures were carried out in 0.6% NaCl, 60% glycerol as the negative control and also the positive control with the addition of the substance with antiparasitic activity, namely albendazole. Each experiment was repeated 3 times. After a 24-h exposure, the culture of nematodes of the genus *Rhabditis* sp. was observed in terms of development, deformity, damage and motility using a stereoscopic and light microscope (Olympus, Tokyo, Japan). To determine the viability of nematodes, they were stained with methylene blue. Live and dead specimens were counted in Bürker (Brand, Wertheim, Grossostheim, Germany) counting chambers.

Statistical analysis was performed using the GraphPad Prism 5.02 software (GraphPad Software, San Diego, CA, USA) using the ANOVA variance analysis and the Tukey test (statistically significant differences were adopted with the coefficient value of *p* < 0.05 *, *p* < 0.01 **, *p* < 0.001 ***) [39,40,41,42].

### 3.4. Molecular Modeling

#### 3.4.1. Protein Preparation

A tubulin structure in complex with colchicine (PDB ID 5NM5 [27]) was selected and prepared using Maestro (Version 12.5.139) [43]. The dimer consisting of chains A (tubulin α-1B) and B (tubulin β-2B) in complex with colchicine and GTP were separated to mimic the closest to the natural binding environment for the docked ligands. The hydrogen atoms were added in the idealized positions. The missing side chains, located a long distance from the colchicine’s binding side, were added using the conformers’ library. Colchicine and water molecules were removed for the next step of the experiment. 

Additional note: the 5NM5 structure is a Bos Taurus tubulin, which shares 88.99% sequence identity with tubulin β-2B from *Caenorhabditis elegans*—the organism closely related to the one used in the biological activity tests. The sequence alignment was obtained with Clustal Omega [44,45]. 

Most of the non-identical residues are a long distance from the colchicines binding site. Only four residues are in the proximity of the binding site, however, the character of the mutation does not indicate strong differences in the plausible interactions. 

#### 3.4.2. Ligands Preparation

The initial 3D geometries of ligands were taken from the crystal structure data. Colchicine randomized 3D structure for the re-docking experiment, as well as the albendazole molecule, wereobtained from SMILES with OpenBabel (version 2.4.1) [46] and their geometries were initially minimized with the MM+ force field in HyperChem Professional 8.0 [47] integrated molecular modelling software.

#### 3.4.3. Molecular Docking Procedure

Docking procedures were conducted in GOLD [48]. The binding site was centred at Leu255 (CD1 atom) and included all residues within the sphere of 10 Å radii, fully covering the colchicine’s residing space. During the docking process, ligands were allowed flexibility to find the most probable conformation (genetic algorithm (GA) implemented in GOLD [48]). Only the chain linking the rhodanine and aromatic moiety was kept rigid due to its nature (conjugation) and to guarantee the fully extended conformation, without alternative isomers consideration. Selected active residues constituting the original colchicines binding site (Cys241, Leu242, Leu248, Asn258, Ile318, Lys352) were treated as flexible during the docking procedure. The selection was adjusted stepwise based on multiple docking procedures and careful inspection of whether the alternative site chains conformations are generated during ligand binding. The empirical ChemScore scoring function [49] was applied to assess the binding affinity.

#### 3.4.4. Log P and Log S Prediction

The log P and log S values for the investigated compounds were predicted using the ALOGPS 2.1 Java applet of the Virtual Computational Chemistry Laboratory (http://www.vcclab.org; calculations have been performed on 3 February 2022) [50]. All obtained results are shown in Table 3.

## 4. Conclusions

New cinnamaldehyde derivatives of rhodanine were synthesized to search for new compounds with anthelmintic activity against *Rhabditis* sp. In the series of 16 compounds, only one shows very high activity, namely 5-(4′-methoxycinnamylidene)-rhodanine-3-etanoic acid (**2**). Anthelmintic activity of compound **2** is higher than that of the commercial drug—albendazole (Figure 5). We set the hypothesis that the mechanism of action of **2** is through the inhibition of microtubule polymerisation, just as it is postulated for albendazole. The molecular modelling studies have shown good binding of selected compounds to the colchicine highly hydrophobic pocket, which is a known albendazole putative binding site. The lack of anthelmintic activity of other investigated compounds can be connected with their higher lipophilicity in comparison to active compound **2** and albendazole (Table 3). It is worth noting that the lipophilicity of albendazole is higher than that of compound **2**, which may explain its lower anthelmintic activity. The mechanism of action of **2** will be verified in the next step of our research. Thus, compound **2** is a good drug candidate with anthelmintic activity.

## Figures and Tables

**Table 1:**
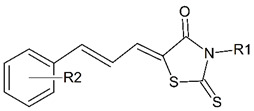
Structures of reported compounds.

Compound	R1	R2	Compound	R1	R2
**1**	H	*p*-OCH_3_	**9**	CH_2_COOH	*o*-OCH_3_
**2**	CH_2_COOH	*p*-OCH_3_	**10**	H	*m*-OCH_3_
**3**	(CH_2_)_2_COOH	*p*-OCH_3_	**11**	CH_2_COOH	*m*-OCH_3_
**4**	(CH_2_)_3_COOH	*p*-OCH_3_	**12**	(CH_2_)_2_COOH	*p*-NO_2_
**5**	(CH_2_)_4_COOH	*p*-OCH_3_	**13**	CH_2_COOH	*p*-Cl
**6**	(CH_2_)_5_COOH	*p*-OCH_3_	**14**	(CH_2_)_2_COOH	*p*-Cl
**7**	(CH_2_)_10_COOH	*p*-OCH_3_	**15**	CH_2_COOH	*p*-N(C_2_H_5_)_2_
**8**	H	*o*-OCH_3_	**16**	(CH_2_)_2_COOH	*p*-N(C_2_H_5_)_2_

**Table 2:** Fitness score and predicted ΔG of ligand–protein complex formation.

Ligand	ChemScore Fitness the Top Result	ChemScore ΔG (kcal/mol)
colchicine	24.60	−25.19
**14**	19.21	−20.49
**2**	18.77	−19.62
**4**	17.93	−19.55
albendazole	14.83	−15.01
**16**	13.48	−19.35

**Table 3:** The selected physicochemical properties predicted for the studied rhodanine derivatives and the reference anthelmintic drug albendazole (colchicine was added only for the properties comparison).

Compound	log P	log S *
colchicine	1.45	−3.89
albendazole	3.22	−4.07
**2**	2.79	−4.86
**4**	3.44	−4.97
**14**	3.63	−5.06
**16**	4.59	−4.87

* Classification of solubility: log S ≤ −10 insoluble; −10 ˂ log S ≤ −6 poorly; −6 ˂ log S ≤ −4 moderately; −4 ˂ log S ≤ −2 soluble; −2 ˂ log S ≤ 0 very; log S ˂ 0 highly.

## Data Availability

All data generated or analyzed during this study are included in this published article. Moreover, the datasets used and/or analyzed during the current study are available from the corresponding author on reasonable request.

## Sample Availability

Samples of the investigated and described compounds are available from the authors.
